# Comparative Effects of Organic and Mineral Calcium Sources on Yield, Nutritional Quality, and Postharvest Storage of Aubergine (*Solanum melongena* L.)

**DOI:** 10.1002/fsn3.72023

**Published:** 2026-06-14

**Authors:** Djabou Mouafi Astride Stéphanie, Boutchouang Pouengue Rodrigue, Batoum Yvan Cedric, Kouam Eric Bertrand, Niemenak Nicolas

**Affiliations:** ^1^ Faculty of Agronomy and Agricultural Sciences University of Dschang Dschang Cameroon; ^2^ Laboratory of Plant Physiology and Biochemistry, Department of Biological Science, Higher Teacher Training College University of Yaoundé 1 Yaoundé Cameroon; ^3^ Department of Sustainable Agriculture and Disaster Management, Faculty of Sciences University of Garoua Garoua Cameroon

**Keywords:** Aubergine, calcium sources, quality, shelf‐life, yield

## Abstract

Aubergine is a horticultural crop valued for its nutritional quality, particularly its phenolic compounds, vitamins, and dietary fiber. However, its production is low due to poor fertilization management and the short shelf life of its fruits. This study aims to evaluate the effects of organic calcium fertilization with eggshell and beef bone powders compared with mineral calcium nitrate fertilization on aubergine yield, storage, and quality. The experimental design was a randomized block design with eight treatments consisting of two types of organic calcium sources for fertilization: eggshell powder and beef bone powder, each at two levels of application (30 g and 60 g per liter per plant); two levels of calcium nitrate application (10 g and 15 g per liter per plant), NPK 20‐10‐10 application at the level of 15 g per liter per plant, and the control treatment (0 g of fertilizer application), each treatment repeated three times. The results indicated that the highest fruit weight (386.11 ± 21.79 g), fruit girth (36.60 ± 1.94 cm), and net yield (66.64 ± 10.82 t/ha) were obtained with the control treatment, while the highest fruit number (7.40 ± 0.54 fruits) was obtained with bone powder_60g. The analysis of fruits during storage showed that treatments with eggshell powder (60 g) and calcium nitrate (15 g) extended the shelf life of fruits up to 30 days, whereas beef bone powder (30 g) resulted in the highest fruit loss rate. The analysis of the nutritional quality of the fruits at harvest showed that the treatments with eggshell powder significantly improved the contents of soluble sugars, polyphenols, proteins, and flavonoids compared to the plants treated with beef bone powder and the control. These findings indicate that eggshell powders can serve as alternative organic calcium sources to improve the nutritional quality and postharvest performance of aubergine fruit.

## Graphical abstract

We assessed the comparative effects of applying organic and mineral calcium sources on aubergine production, quality, and storage. Analysis of yield parameters, nutritional quality of fruits at harvest and fruits physical quality during storage showed that the use of eggshell and beef bone powders as calcium sources can improve aubergine fruit production, quality, and storage, while providing an alternative to mineral calcium.

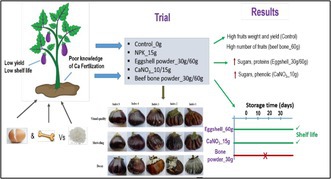

## 1 Introduction

Aubergine ( *Solanum melongena* L.), also known as “Guinea gourd” fruit, is a non‐tuberous species belonging to the Solanaceae family (Kantharajah and Golegaonkar 2004). Aubergine is highly cultivated in Asia and some Mediterranean countries. The global aubergine production was estimated at 6079 thousand tons in 2023 (FAOSTAT 2023). Its fruits are highly appreciated for their good nutritional value and therapeutic properties (Singh et al. 2009; El‐Nemr et al. 2012; Sharma and Kaushik 2021; Colak et al. 2022). Aubergine is a source of income generation especially at a specific time of the year when other sources of employment or income are limited (Verheij and Henk 2008). Then, the increasing consumption of aubergines, both in its natural form and as dry extract capsules, presents an optimistic scenario for the crop's expansion. However, soil fertility decline and excessive use of mineral fertilizers are major constraints of aubergine production (Valenzuela et al. 2017). Furthermore, rapid population growth is leading to increased food waste, including eggshells, animal carcasses, and bone meal. The high concentration of calcium in eggshell and bone meal from waste may represent an alternative to mineral fertilizers, which are very expensive and harmful to humans and the environment (Suge et al. 2011). Calcium plays a crucial role in plant physiology and stress adaptation. It is implicated in the elongation and division of plant cells, as well as in the regulation of cell membrane permeability, nitrogen metabolism, and carbohydrate translocation (White 2000; El Beltagi and Mohamed 2013). Additionally, it reduces fruit rot and increases firmness and shelf life (Al Eryani‐Raqeeb et al. 2009; Coulibaly et al. 2023). The work of Aghofack et al. (2014) has shown that applying calcium nitrate at 200 kg/ha to tomatoes reduced the time from field transplanting to fruit formation and storage.

However, poor fertilizer management, particularly of calcium, leads to a nutritional imbalance in plants, resulting in the production of inferior‐quality fruit unsuitable for storage and the market (Valenzuela et al. 2017). Recent studies on calcium application have shown that foliar application of calcium nitrate and calcium chloride have significantly increased fruit growth and yield in peppers (Swelam and Abd El‐Basir 2021). Similarly, eggshell, bone meal, and snail powders have been reported as organic sources of calcium to improve growth, yield, and reduce apical necrosis in tomatoes (Coulibaly et al. 2023; Dekoum and Petang 2024). Even if various works evaluated the performance of aubergine fruit during storage and under NPK fertilization (Fallik et al. 1995; Concellón et al. 2007; Concellón et al. 2012; Maylani et al. 2024), still, no one has examined the application of organic calcium based on eggshell/beef bone on fruit production, quality, and storage. The objective of this study was to assess the effect of organic calcium fertilization from eggshell powder and beef bone powder compared to mineral fertilization with calcium nitrate on yield, quality, and storage of aubergine fruits. This work would like to introduce a novel approach by assessing locally sourced organic calcium fertilizers derived from eggshell and beef bone powders as sustainable alternatives to synthetic calcium nitrate and common NPK fertilizers. Eggshells and beef bones are abundant, low‐cost waste materials, making calcium fertilization accessible to smallholder farmers. The transformation and the use of these wastes into valuable inputs may reduce reliance on mineral fertilizers while addressing calcium deficiencies, which are common in tropical soils like those in Cameroon. In addition, the conversion of these food industry waste into crop nutrients respects the principles of the circular economy.

## 2 Materials and Methods

### 2.1 Experimental Site

A field experimentation was conducted at the experimentation Farm of the Faculty of Agronomy and Agricultural Sciences—Annex of Bafia, University of Dschang, Cameroon, in September 2024. The locality of Bafia, Center Region, Cameroon (4°45′00″ N and 11°14′00″ E, 495 m altitude) is an agroecological zone known as the wet rainforest and characterized by bimodal rainfall patterns. The annual precipitation totaled 408 mm in the first 3 months, while a much higher amount (2320 mm) fell from January to April. The soil of the experimental site is a typical clay loam texture, showing the following mineral characteristics: pH in water (4.45), organic matter 7.62 (%); *N* (%) = 0.13; Mg^2+^; Ca^2+^; Na^+^ (cmol/kg) = 1.24; 3.46; 0.60; besides P (mg/kg) and K (cmol/kg) = 32.75 and 0.58, respectively.

### 2.2 Plant Material and Experimental Design

The seeds of aubergine, variety Black beauty, were purchased from certified agro‐input suppliers in Dschang, and sown in nursery beds. This variety was selected for its earliness, adaptability to hot climates, and resistance to water stress. The soil was enriched with poultry manure at 1 kg/m^2^ and urea and NPK (20‐10‐10) at 70 g/m^2^, according to Aghofack et al. (2014). Seedlings in the nursery were treated with the fungicide Ivory 80 (80% mancozebe) after every rainfall. The eggshells and beef bones, common waste products from fast‐food restaurants and slaughterhouses, respectively, were collected in Bafia, Cameroon's Center region. Eggshells were thoroughly cleaned with distilled water, surface‐sterilized by immersion in hot water, sun‐dried (30°C–34°C) for 3 days, and then ground into a homogeneous fine powder using a grinder (Ishita et al. 2022). After sun‐drying, beef bones were heated in a metal drum until a gray ash formed, then ground into a fine, homogeneous powder using a grinder (Razafindramanana et al. 2020). Table 1 presents the mineral composition of eggshell and bone powders.

**TABLE 1:** Mineral composition of eggshell and beef bone powders used as organic calcium sources of fertilization.

Calcium source	Mineral composition					
	N (mg/kg)	P (mg/kg)	K (mg/kg)	Ca (mg/kg)	Mg (mg/kg)	Na (mg/kg)
Eggshell powder	0.74	12.49	454.23	3267.00	799.80	32.98
Bone powder	0.68	11.30	453.01	1361.50	656.55	297.25

The experimental design was a randomized block design with eight treatments consisting of two types of organic calcium sources for fertilization: eggshell powder and beef bone powder each at two levels of application (30 g, 60 g per liter per plant equivalent of 0.8 t/ha and 1.6 t/ha, respectively) according to Anagrah et al. (2021) modified, Dekoum and Petang (2024) and Coulibaly et al. (2023) modified; two levels of calcium nitrate application (10 g and 15 g per liter per plant equivalent of 0.277 t/ha and 0.417 t/ha, respectively) considered as a mineral calcium source according to Seifu and Deneke (2017) modified, NPK application at the level of 15 g per liter per plant (equivalent of 0.417 t/ha) considered as standard fertilization commonly used by farmers and the control treatment (0 g of fertilizer application). The treatments were administered 3 weeks after transplanting. The seedlings were planted at a depth of 2 cm and watered immediately after transplanting. Plants were spaced 60 cm apart, with 20 plants per plot measuring 2 m × 5 m. Weeds were removed manually as soon as they appeared to avoid competition with aubergines for soil nutrients.

### 2.3 Assessment of Yield Parameters

Harvesting took place 7 weeks after planting. The aubergine fruits were harvested and stored in a cool, dry place. Thirty plants were randomly selected per treatment to evaluate yield parameters, including the number of fruits per plant through manual counting, fruit length (cm), fruit girth (cm) using a swining meter, fruit weight (g) using an electronic balance and average yield (t/ha). The total yield was calculated according to Coulibaly et al. (2023) using the following formula:

$$Y\left(t/\mathrm{ha}\right)=\frac{\;\text{Fruit mass of}\;a\;\text{given experimental unit}}{\text{Total surface of the experimental unit}}$$

After harvest, 45 fruits from each treatment were selected for the postharvest experiment based on their uniformity in size and color. Fruits were carefully observed daily for 30 days to evaluate the decrease in fruit quality during storage at room temperature.

### 2.4 Assessment of Fruit Nutritional Quality at Harvest

#### 2.4.1 Determination of Sugar and Protein Contents

Total soluble sugars were extracted following a modified anthrone method as described by Dubois et al. (1951). Fruit flesh and pulp (200 mg) were ground in a mortar with 4 mL of 80% (v/v) ethanol, then centrifuged at 3500 *g* for 10 min. The supernatant was collected and constituted the crude extract. An aliquot of 15 μL of this alcoholic extract was added to 2.5 mL of Anthron reagent, homogenized, and incubated at 80°C for 20 min. After cooling in melting ice, the absorbance of the green complex was determined at 620 nm, using glucose as a standard.

Total protein quantification was assessed at harvest from 500 mg of fruit, which was ground in a chilled mortar with 4 mL of TAMET buffer (0.5 M Tris, 0.3 M ascorbic acid, 0.2% (v/v) β‐mercaptoethanol, 0.01 M EDTA, and 0.02% (v/v) Triton X‐100, pH 6.7). The crude homogenate was centrifuged at 20,000 × *g* for 15 min at 4°C. The supernatant was collected and used as the crude protein extract. Total protein concentration was estimated using the Bradford method (Bradford 1976). Bradford reagent (2 mL) was added to 50 μL of protein extract and 185 μL of phosphate buffer. The mixture was incubated for 2 min, and the absorbance of the blue‐colored complex was measured at 595 nm. Bovine serum albumin (BSA 0.5–60 μg/μL) served as the standard.

#### 2.4.2 Determination of Total Phenolic and Flavonoids Contents

Total phenolic compound content in fruit was extracted as described by Boizot and Charpentier (2006). Fresh fruit tissue (500 mg) was ground in 5 mL of 80% (v/v) methanol at 4°C, then centrifuged three times at 7000 *g* for 30 min, and the supernatant was recovered each time. A mixture of the three supernatants constituted the crude phenolic compounds extract, quantified using the method described by Singleton and Rossi (1965). An aliquot of 15 μL of alcoholic extract was added to 250 μL of Folin–Ciocalteu reagent, 2.5 mL of distilled water, and 0.5 mL of sodium carbonate (20%). The mixture was incubated at 40°C for 20 min, and the blue color was determined at 760 nm using a spectrophotometer. A calibration standard curve was established using gallic acid in 80% ethanol, and the result was expressed in mg of gallic acid equivalent per fresh weight (mg GAE g/FW).

The polyphenol extract was mixed with formaldehyde at a 1:1 (v/v) ratio to allow flavonoids to precipitate. The flavonoids were removed from the mixture by filtration, and the non‐flavonoid extract was assayed by the method of Singleton and Rossi (1965). The total flavonoid content of the extracts was expressed in mg gallic acid equivalent per g of fresh matter (FW). It was calculated according to Singleton et al. (1999) using the following formula:

$$\left[\text{Flavonoid}\right]=\left[\text{Total polyphenols}\right]-\left[\text{non}-\text{flavonoid}\right]$$

### 2.5 Assessment of Fruit Quality During Storage

#### 2.5.1 Weight Loss

Harvested fruits were taken to the laboratory, grouped by treatment, cleaned, and placed on a bench at 25°C ± 5°C and 60% ± 3% RH. Fruits were analyzed at 0, 5, 10, 20, 25, and 30 days of storage. Individual fruits were weighed before storage. Twenty fruits were evaluated for each treatment and storage time. The percentage of weight loss (WL) was calculated as follows:

$$\%\text{Weight loss}\;\left(\mathrm{WL}\right)=\frac{w_{i}-w_{f}}{w_{f}}\times 100$$

*w*_*i*_ is the initial sample weight, and *w*_*f*_ is the final sample weight at a specific time. Results were expressed as a percentage of weight loss.

#### 2.5.2 Mechanical Strength Loss

The loss of mechanical strength (loss of firmness) in fruits was measured using a crossbow‐type penetrometer on the median part of the fruit and analyzed as described by Lepengue et al. (2012). It was calculated at 0, 5, 10, 15, and 20 days after harvest by analogy with an equation based on the initial and final resistances.

### 2.6 Assessment of Physical Quality of Fruit During Storage

Visual quality, Shriveling, and the degree of degradation were evaluated on 20 fruits at 0, 5, 10, 15, 20, 25, and 30 days after harvest (DAH), according to Concellón et al. (2012) and Bayogan et al. (2017). Visual quality rating was defined as follow: index 5 = excellent, fresh appearance; index 4 = very good, slight defects; index 3 = good, limit of saleability, defects progressing; index 2 = fair, usable but not saleable; index 1 = poor, the shriveling index were defined as follow: index 5 = no Shriveling; index 4 = slight, 1%–15% of surface area shriveled; index 3 = moderate, 16%–30% of surface area shriveled; index 2 = severe, 31%–49% of the surface area shriveled; index 1 = extreme, ≥ 50% of the surface area shriveled and the degree of degradation was defined as follow: index 5 = no decay; index 4 = 1%–10% decay/slight; index 3 = 11%–25% decay/moderate; index 2 = 26%–50% decay/moderately severe; index 1 = more than 50% decay/severe.

### 2.7 Statistical Analysis

Microsoft Excel was used to summarize raw data and prepare figures to facilitate a more thorough evaluation of the results. Moreover, a comparison of the effects of different treatments was performed using analysis of variance. Finally, the means of the different treatments were separated using the Student–Newman–Keuls and Duncan tests (*p* = 0.05).

## 3 Results

### 3.1 Fruits Yield Parameters

Fruit yield parameters were significantly affected by the source of calcium application (Table 2). The highest number of fruits per plant (7.40 ± 0.54) was recorded in treatment T7, while the lowest was recorded in treatment T4 (4.60 ± 0.54). Regarding fruit weight, the T0 treatment showed the highest (386.11 ± 21.79 g), followed by the bone powder treatments, which recorded the lowest fruit weight: 287.43 ± 15.49 g for T6 and 182.22 ± 11.85 g for T7, respectively. No significant difference was observed in terms of fruit length across the treatments. However, a significant difference was observed across treatments in the average fruit girth. The results showed that plants from the T0 treatment produced the highest fruit girth (36.60 ± 1.94 cm) compared to the lowest fruit girth (28.20 ± 0.83 cm) observed in the treatment T7. The best net yield (66.64 ± 10.82 t/ha) was obtained in the T0 treatment, while the lowest net yield (37.33 ± 5.10 t/ha) was recorded in the treatment T4. Meanwhile, no significant differences were found between the treatments T4 and T7.

**TABLE 2:** Yield parameter of eggplant treated with chemical and organic source of calcium at seven weeks after planting.

Codes	Treatments	Fruit number	Fruit length (cm)	Fruit girth (cm)	Fruit weight (g)	Fruit yield (t/ha)
T0	Control_0g	6.20 ± 0.84 b	11.60 ± 0.41 a	36.60 ± 1.94 a	386.11 ± 21.79 a	66.64 ± 10.82 a
T1	NPK_15g	5.80 ± 0.84 bc	11.20 ± 1.09 a	31.20 ± 0.44 b	315.94 ± 15.52 b	50.75 ± 6.25 b
T2	Eggshells powder_30g	5.20 ± 0.45 bc	11.00 ± 0.70 a	30.80 ± 0.44 bc	310.38 ± 5.78 bc	44.84 ± 4.11 bc
T3	Eggshells powder_60g	5.20 ± 0.45 bc	12.10 ± 1.14 a	31.20 ± 0.83 b	324.40 ± 23.89 b	46.77 ± 4.14 b
T4	Calcium nitrate_10g	4.60 ± 0.55 c	11.60 ± 0.54 a	31.00 ± 1.41 b	291.78 ± 12.79 c	37.33 ± 5.17 c
T5	Calcium nitrate_15g	5.00 ± 0.71 bc	11.80 ± 0.83 a	29.20 ± 0.44 cd	306.47 ± 18.65 bc	42.68 ± 7.79 bc
T6	Bones powder_30g	5.40 ± 1.14 bc	10.60 ± 0.82 a	29.60 ± 0.89 bcd	287.43 ± 15.49 c	42.79 ± 7.19 bc
T7	Bones powder_60g	7.40 ± 0.55 a	11.40 ± 0.54 a	28.20 ± 0.83 d	182.22 ± 11.85 d	37.37 ± 2.32 c

*Note:* Means followed by the same letter in the same column are not significantly different at the 5% level according to Student–Newman–Keuls test.

### 3.2 Fresh Fruits' Nutritional Quality at Harvest

The nutritional composition of fresh aubergine fruit with varying sources of calcium fertilization is presented in Table 3. The analysis of total soluble sugars revealed a higher accumulation of sugars in fruits from plants treated with eggshell powder compared to other treatments. No significant difference in sugar content was observed for the plant treated with calcium nitrate at T4 and T5. In addition, the application of different sources of calcium fertilization had a positive effect on the total protein content, as the fruits from the treatment with no application present the lowest total protein content compared to those from plants treated with the different sources of calcium. Fruits from plants treated with calcium nitrate at T4 presented the highest total phenolic compounds (1.66 ± 0.02 mg g^−1^ FW) accumulation.

**TABLE 3:** Nutritional analysis of eggplant fruits at harvest from the different treatments.

Codes	Treatments	Proteins content (mg g^−1^ FW)	Sugars content (mg g^−1^ FW)	Phenolics content (mg g^−1^ FW)	Flavonoids content (mg g^−1^ FW)
T0	Control_0g	3.73 ± 0.08 d	13.19 ± 1.81 ab	1.50 ± 0.04 c	0.85 ± 0.03 ab
T1	NPK_15g	4.16 ± 0.06 bc	15.22 ± 3.68 ab	1.39 ± 0.07 de	0.69 ± 0.05 b
T2	Eggshells powder_30g	4.69 ± 0.05 a	15.89 ± 0.89 a	1.59 ± 0.01 b	0.87 ± 0.06 ab
T3	Eggshells powder_60g	4.45 ± 0.11 ab	16.32 ± 2.18 a	1.50 ± 0.02 c	0.73 ± 0.03 ab
T4	Calcium nitrate_10g	4.59 ± 0.22 a	11.68 ± 2.94 b	1.66 ± 0.02 a	0.80 ± 0.07 ab
T5	Calcium nitrate_15g	3.78 ± 0.13 d	11.78 ± 0.54 b	1.33 ± 0.06 e	0.41 ± 0.04 c
T6	Bones powder_30g	4.47 ± 0.25 ab	11.44 ± 1.75 b	0.90 ± 0.03 f	0.22 ± 0.02 c
T7	Bones powder_60g	3.94 ± 0.09 cd	12.39 ± 1.19 ab	1.43 ± 0.03 cd	0.97 ± 0.40 a

*Note:* Means followed by the same letter in the same column are not significantly different at the 5% level according to Student–Newman–Keuls test.

The lowest total phenolic compounds (0.90 ± 0.03 mg g^−1^ FW) were recorded in fruits from plants treated with bone powder at T7. The flavonoid contents of aubergine fruits were determined in phenolic extracts. Fruits from T7 exhibited the highest flavonoid content (0.97 ± 0.40 mg g^−1^ FW). Regarding total phenolic compounds, a very low flavonoid content was recorded in fruits from T6. No statistical differences in flavonoid content were found among the treatments T0, T2, and T3.

### 3.3 Change in Physical Quality of Fruits During Storage

#### 3.3.1 Fruits' Weight Loss and Firmness Loss

**FIGURE 1:**
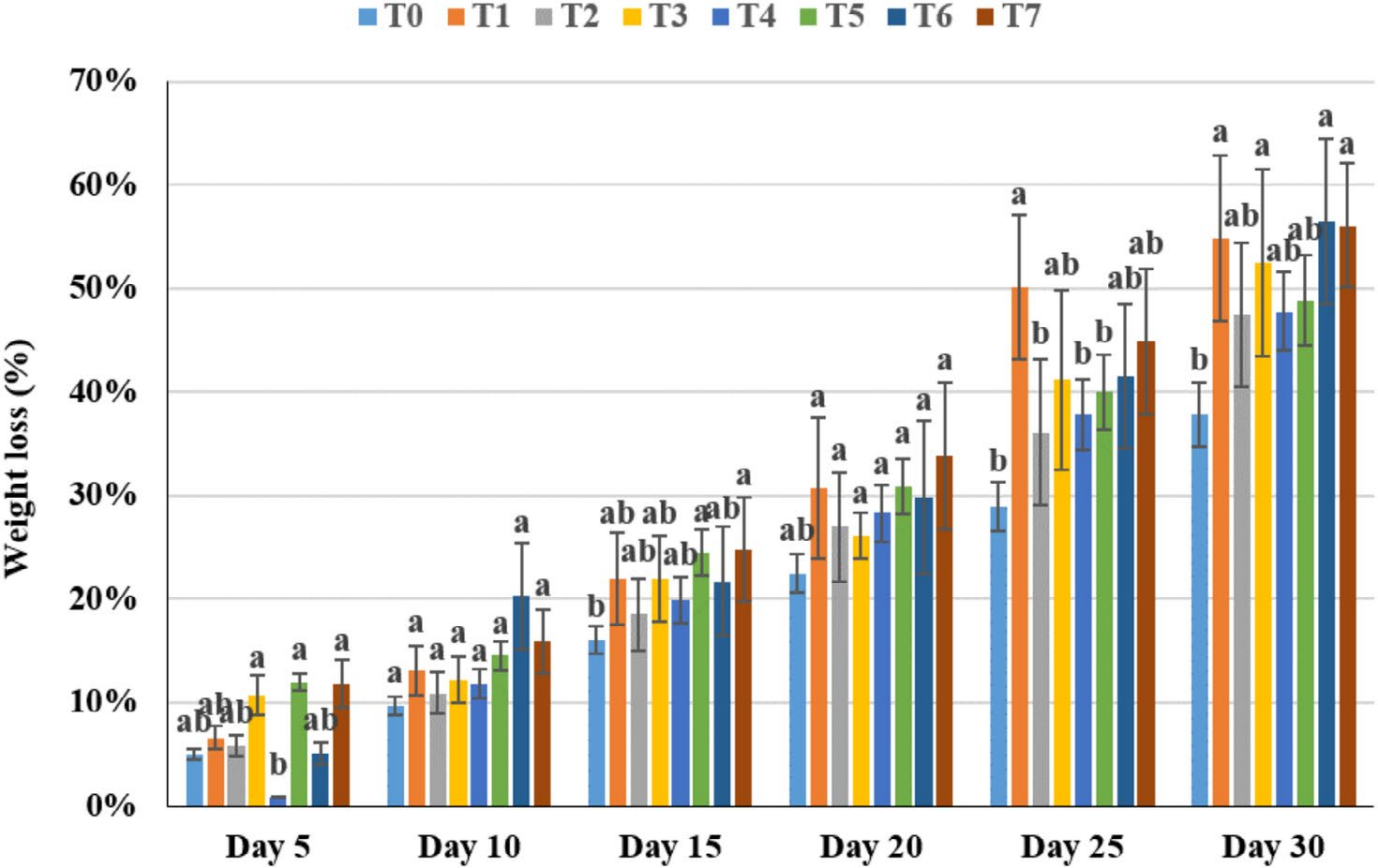
Effect of treatments on fruits weight loss during storage. Mean separations were performed with Duncan's significant difference at *p* = 0.05 and values followed by the same letter at each time point are not significantly different. T0 = control_0g, T1 = NPK_15g, T2 = eggshell powder_30g, T3 = eggshell powder_60g, T4 = calcium nitrate_10g; T5 = calcium nitrate_15g, T6 = beef bones powder_30g; T7 = beef bones powder_60g.

The analysis of weight loss of aubergine fruits showed a significant variation (*p* ≤ 0.05) during the 30 days' storage period (Figure 1). An increase in weight loss was observed in all the treatments during storage. However, high fruit weight loss was observed for treatments T7 (35.92%) and T3 (34.39%) compared to T0 (27.43%) at the end of 30 days of storage. Regarding the loss of firmness, the treatments T2 and T3 showed less loss of fruit firmness than the other treatments at 20 days of storage (Table 4).

**TABLE 4:** Loss of firmness (%) of eggplant fruits during storage at room temperature.

Codes	Treatments	0 DAH	5 DAH	10 DAH	15 DAH	20 DAH
T0	Control_0g	6.18 ± 0.28cA	6.19 ± 0.67dA	6.08 ± 0.81bcA	5.01 ± 0.47cA	5.89 ± 0.15cA
T1	NPK_15g	8.02 ± 0.76abA	7.92 ± 0.18abA	7.39 ± 0.23aAB	6.31 ± 0.13bcB	6.85 ± 0.97bcAB
T2	Eggshells powder_30g	5.37 ± 0.59c A	6.86 ± 0.21bcdA	6.92 ± 0.22abA	3.68 ± 1.44 dB	3.93 ± 0.70eB
T3	Eggshells powder_60g	7.87 ± 0.60abA	6.58 ± 0.27cdB	7.70 ± 0.31aA	7.29 ± 0.35abAB	4.88 ± 0.41dC
T4	Calcium nitrate_10g	8.61 ± 0.49aA	8.06 ± 0.89abA	6.14 ± 0.14bc B	6.09 ± 0.32bcB	6.05 ± 0.05cB
T5	Calcium nitrate_15g	7.41 ± 0.37bA	7.58 ± 0.51abcA	5.28 ± 0.85cdB	7.01 ± 0.02abA	7.45 ± 0.44abA
T6	Bones powder_30g	7.23 ± 0.34bB	8.38 ± 0.51aA	4.56 ± 0.04dC	5.18 ± 0.45cC	8.17 ± 0.70aA
T7	Bones powder_60g	5.57 ± 0.40cB	6.98 ± 0.33bcdA	5.23 ± 0.83cdC	7.70 ± 0.04aA	7.57 ± 0.15abA

*Note:* Means followed by the same lower‐case in the same column and means followed by the same upper‐case letters in the same ligne are not significantly different at the 5% probability level according to Student–Newman–Keuls test.

Abbreviation: DAH, day after harvest.

#### 3.3.2 Visual and Commercial Fruits Quality

**FIGURE 2:**
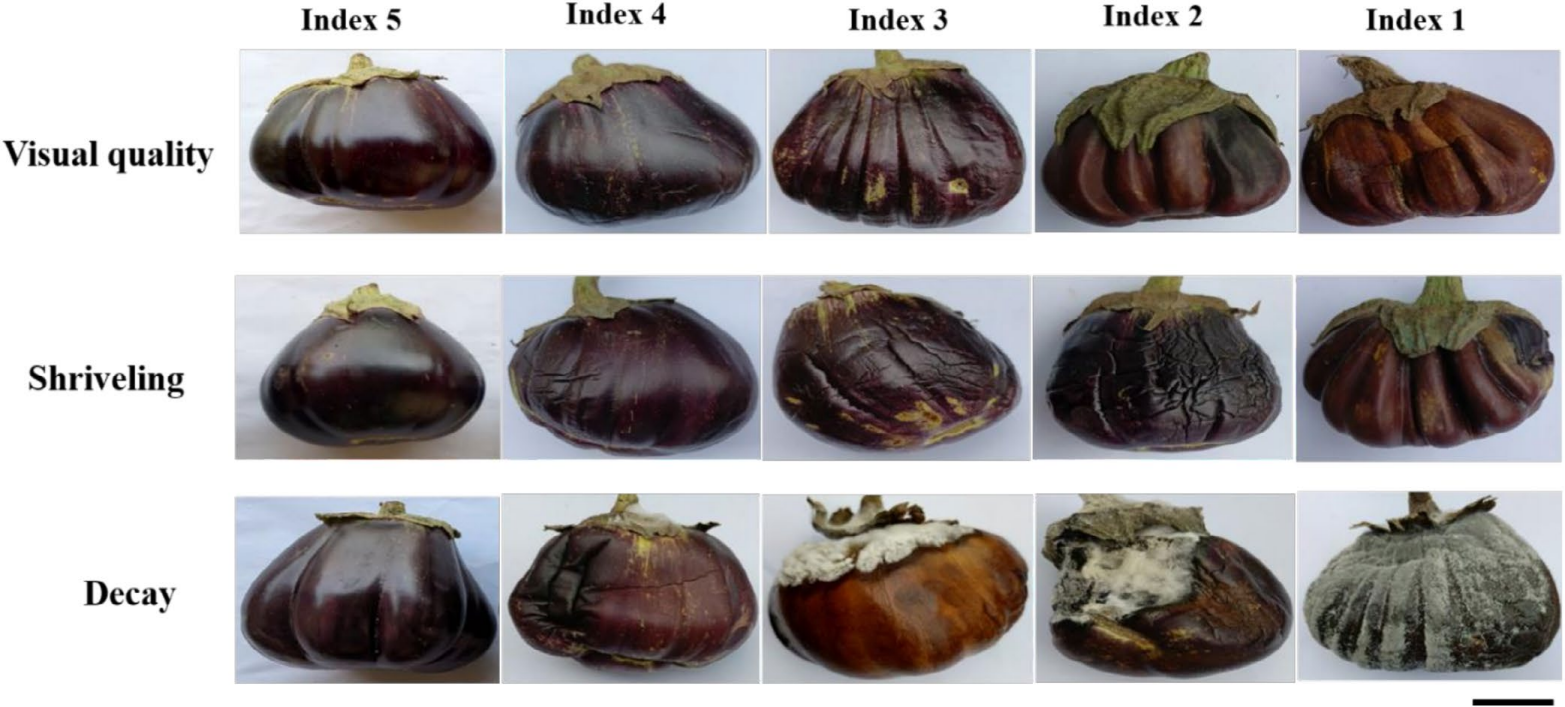
Rating scale for the appreciation of physical quality of fruits during storage. Visual quality rating (Index 5 = excellent, fresh appearance; Index 4 = very good, slight defects; Index 3 = good, limit of saleability, defects progressing; Index 2 = fair, usable but not saleable; Index 1 = poor), for shriveling (Index 5 = no shriveling; Index 4 = slight, 1%–15% of surface area shriveled; Index 3 = moderate, 16%–30% of surface area shriveled; Index 2 = severe, 31%–49% of the surface area shriveled; Index 1 = extreme, ≥ 50% of the surface area shriveled) and degree of decay (Index 5 = no decay; Index 4 = 1%–10% decay/slight; Index 3 = 11%–25% decay/moderate; Index 2 = 26%–50% decay/moderately severe; Index 1 = more than 50% decay/severe). Bar = 5 cm.

**FIGURE 3:**
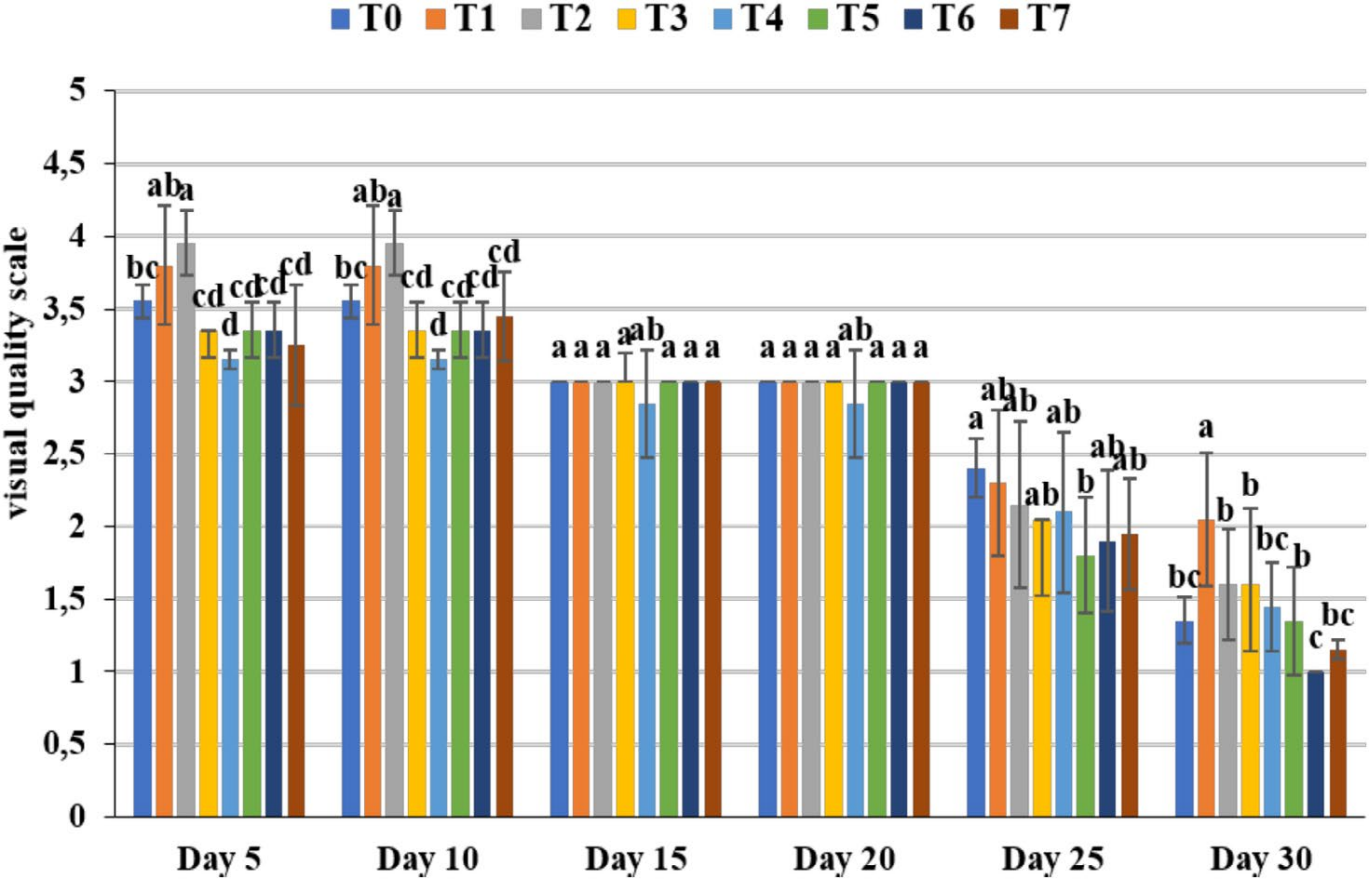
Effect of treatment on fruit visual quality rating scale during storage. Mean separations were performed with Duncan's significant difference at *p* = 0.05 and values followed by the same letter at each time point are not significantly different. T0 = control_0g, T1 = NPK_15g, T2 = eggshell powder_30g, T3 = eggshell powder_60g, T4 = calcium nitrate_10g; T5 = calcium nitrate_15g, T6 = beef bones powder_30g; T7 = beef bones powder_60g.

The rating scale and visual assessment of the visual quality, shrivelling, and decay of aubergine fruits from plants treated with various calcium sources are presented in Figure 2. The visual quality of aubergine fruits varies with treatment and storage period (Figure 3). In general, the commercial aspect of fruit was maintained for plants treated with eggshell powder and for the control treatments. No significant change was observed in the fruit aspect from 0 to 20 DAH. At 25 DAH, the most acceptable quality of fruits was maintained by fruit from the treatment T5, while those from the treatment T7 presented the least acceptable quality. About 40% of fruits acceptable for market were still recorded at 30 DAH for fruits from plants in the treatment T0, and about 20% for fruits from treatments T2, T3, and T5.

#### 3.3.3 Fruits Shriveling and Decay

**FIGURE 4:**
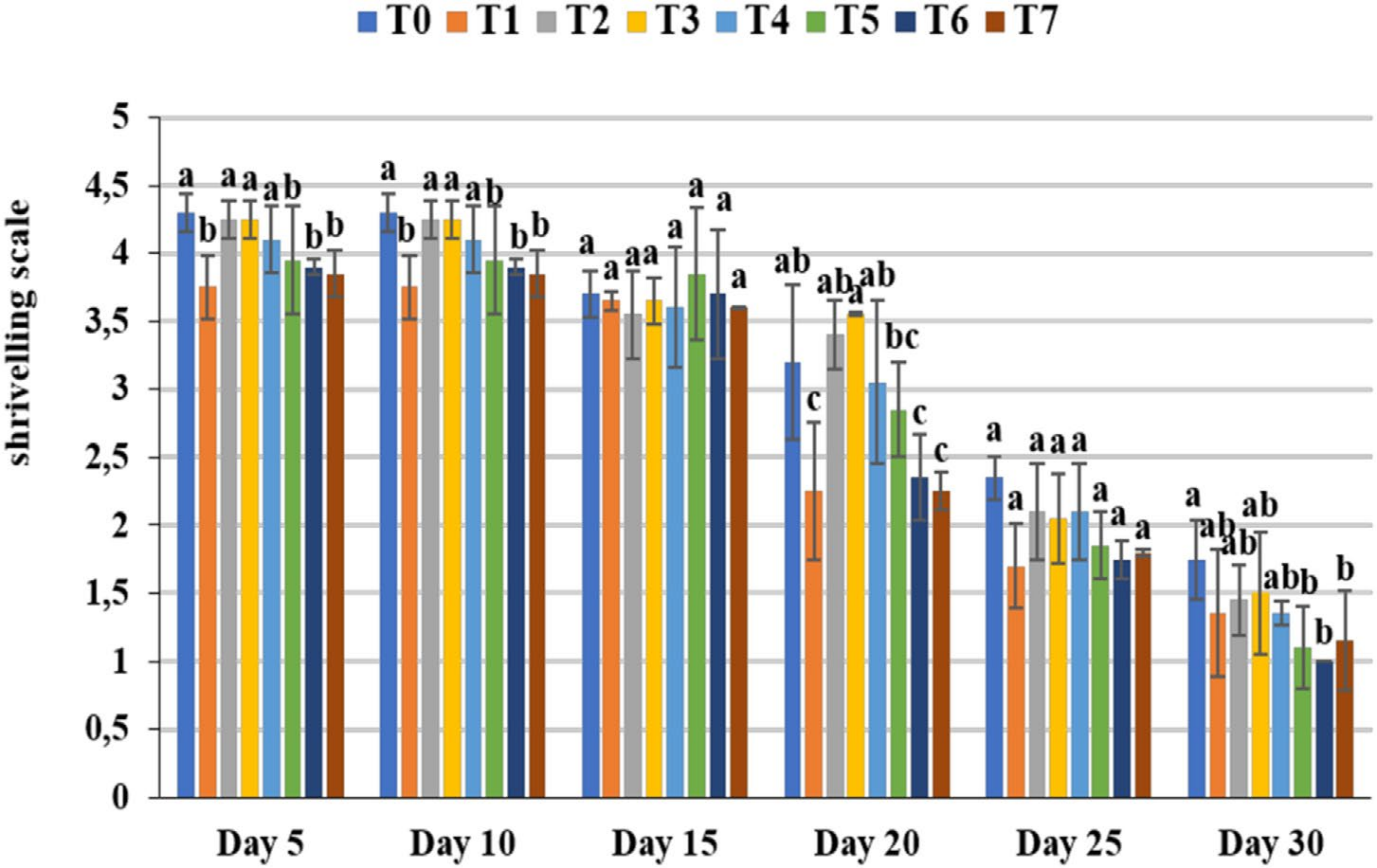
Effect of treatment on fruit shriveling rating scale during storage. Mean separations were performed with Duncan significant difference at *p* = 0.05 and values followed by the same letter at each time point are not significantly different. T0 = control_0g, T1 = NPK_15g, T2 = eggshell powder_30g, T3 = eggshell powder_60g, T4 = calcium nitrate_10g; T5 = calcium nitrate_15g, T6 = beef bones powder_30g; T7 = beef bones powder_60g.

**FIGURE 5:**
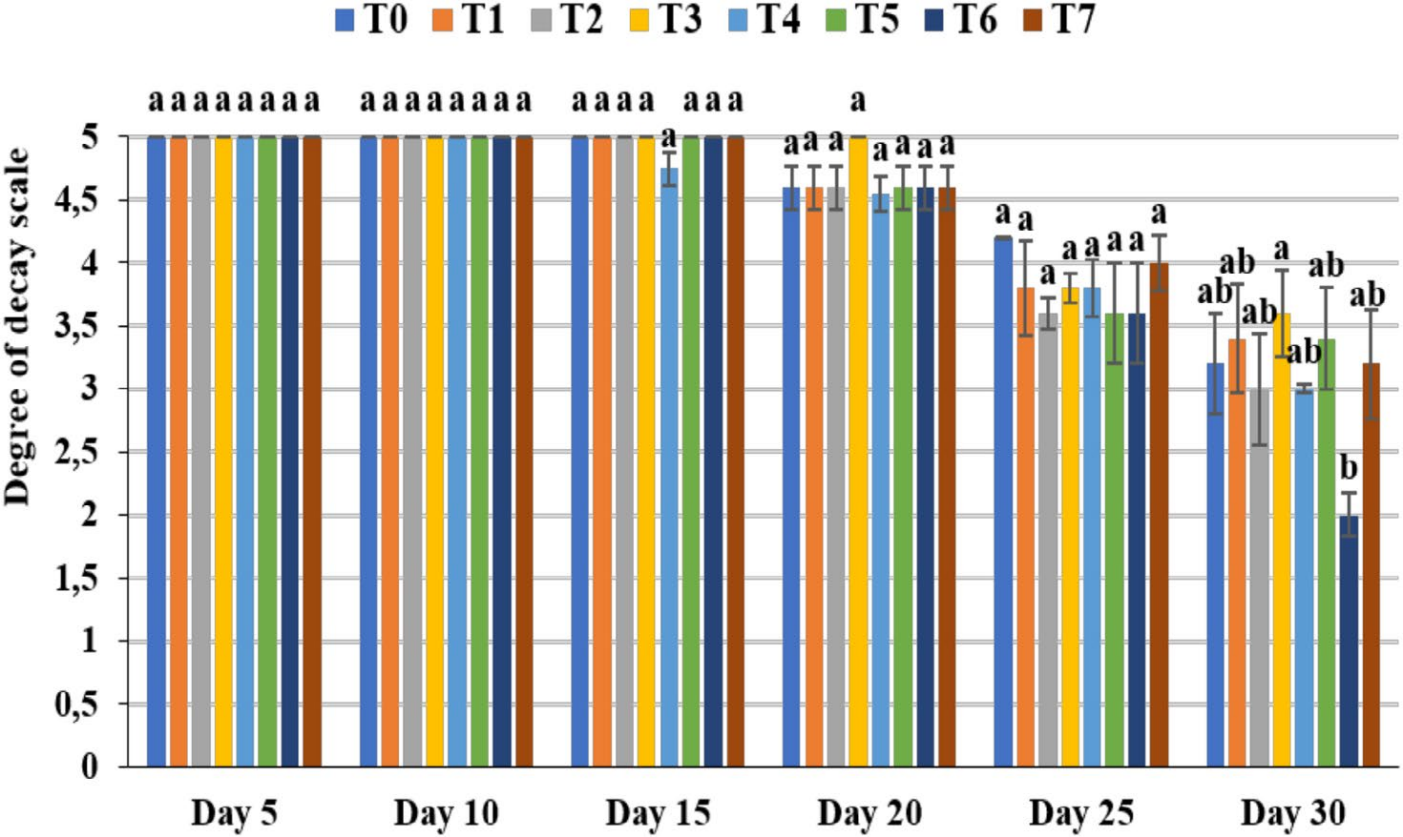
Effect of treatment on fruit degree of decay rating scale during storage. Mean separations were performed with Duncan's significant difference at *p* = 0.05 and values followed by the same letter at each time point are not significantly different. T0 = control_0g, T1 = NPK_15g, T2 = eggshell powder_30g, T3 = eggshell powder_60g, T4 = calcium nitrate_10g; T5 = calcium nitrate_15g, T6 = beef bones powder_30g; T7 = beef bones powder_60g.

The degree of aubergine fruit shrivelling varies with treatment and storage period (Figure 4). No significant change was observed in fruit shrivelling from 0 to 10 DAH. Fruits from treatments T2, T3, and T5 shriveled more slowly than those from T0. About 75% of fruits with moderate shrivelling were still recorded at 30 DAH in T0, while only 8% in T4. Concerning the degree of decay, the treatments T4 and T6 presented the earliest fruit decay and the highest percentage of fruit decay as compared to fruits from the treatment T0 (Figure 5). Furthermore, based on the visual symptoms, anthracnose was observed near the fruit base in the treatment T6 at 30 g after 30 days of storage.

## 4 Discussion

Calcium plays a crucial role in maintaining the overall quality of horticultural crops, particularly fruits and vegetables. It is considered as the main element that determines the structure of cell walls and stabilizes cell membranes (Berridge et al. 2000). It also directly influences the salt balance in plant cells. Furthermore, it is involved in water movement in plants by activating potassium salts, which regulate stomatal movement (Seifikalhor et al. 2019). The analysis of yield variables of aubergine fruit from plants treated with organic or mineral calcium fertilizers did not show significant differences in average fruit size per plant. Significant differences (*p* < 0.05) were observed in average fruit number, fruit weight, fruit girth, and net yield per hectare across treatments. High fruit girth, weight, and yield were recorded in the control treatment with no fertilization, followed by the treatments with eggshell powder. This unexpected superiority of the unfertilized control suggests that moderate or inherent soil fertility or microbial activity in the experimental site may be optimal for fruit development, potentially enhanced by natural nutrient cycling (Smith et al. 2020). In addition, the level of Ca (3.46 cmol/kg) recorded from the soil analysis may imply no acute Ca deficiency. However, the analysis soil showed a pH of 4.45, which can limit nutrient availability, especially phosphorus and potassium. The eggshell powder, rich in calcium more than twice that of bone powder, may likely contribute through improved cell wall strength and nutrient availability, as calcium is crucial for fruit expansion and firmness. In fact, improved nutrient assimilation positively affects photosynthesis and, therefore, plant growth and yield (Lavanya and Bahadur 2021a, 2021b). Studies on similar calcium amendments report up to 20% increases in fruit size due to enhanced Ca uptake (White and Broadley 2003). The observed differences between organic calcium fertilization using eggshell powder and beef bone powder are likely attributable primarily to differences in nutrient composition and release dynamics, with only a minor contribution from alterations in soil physical structure. Eggshell powder is composed predominantly of calcium carbonate (CaCO_3_), typically accounting for more than 90% of its dry weight, and therefore serves mainly as a calcium source and liming material capable of modifying soil pH, cation exchange balance, and nutrient availability (King'ori 2011; Waheed et al. 2020). Because eggshells contain relatively low concentrations of phosphorus (P) and nitrogen (N), their agronomic effects are generally associated with Ca supplementation and pH buffering rather than broad nutrient enrichment (King'ori 2011). In contrast, beef bone powder (bone meal) provides not only calcium but also appreciable quantities of sodium, phosphorus and trace minerals, which can more strongly stimulate root growth, nutrient uptake efficiency, and overall crop productivity (Habtegebrial et al. 2007; Warren et al. 2009) from our analysis, high amount of sodium was observed in beef bone powder analysis that eggshell powder. In fact, At low to moderate concentrations, sodium can partially substitute for potassium in certain physiological functions, including osmotic regulation, stomatal activity, and maintenance of cell turgor, thereby enhancing water uptake and vegetative growth in some crops (Subbarao et al. 2003). In addition, the slower mineralization rate of bone meal can contribute to a more sustained nutrient release pattern compared with eggshell‐derived amendments (Warren et al. 2009). Furthermore, the application rates commonly used for eggshell and bone powders in agricultural systems are generally insufficient to induce substantial modifications in soil physical structure or mechanical aggregation. Their effects are therefore more closely related to nutrient supply, biochemical interactions, and pH regulation than to direct restructuring of the soil matrix (Weil and Brady 2017).

In contrast, bone powder at 60 g per plant yielded the lowest fruit girth, weight, and overall hectare yield, despite producing the highest fruit number per plant. This trade‐off indicates resource dilution, where phosphorus and nitrogen from bone powder (which release slowly during decomposition) promoted prolific flowering and fruit set but limited individual fruit size, possibly due to sink competition or suboptimal C:N ratios that delayed maturation (Marschner 2012). These findings underscore the context‐specific efficacy of organic fertilizers: while eggshell supports balanced fruit quality, bone powder excels in quantity but at a yield cost. Future trials should explore more application rates, soil pH interactions, and combination treatments to optimize both metrics. The bone powder treatment showed the lowest fruit girth, weight, and yield, while the highest fruit number (5 fruits) per plant was observed with the bone powder treatment at 60 g. This number was higher than that reported by Tchiaze et al. (2025), with the same cultivar, using palm nut fiber ash as a calcium source during fertilization. Daşgan and Abak (2003) have shown that plants with high fruit number tend to have low fruit weight and vice versa.

The nutritional analysis of fruits from the different treatments at harvest showed a considerable increase in protein content in fruits from plants treated with calcium fertilizers. The increase in calcium‐hormone interactions undoubtedly influenced hormone signaling, as calcium is a secondary messenger. Calcium is known to participate in gibberellin acid, auxin, and abscisic acid signaling to regulate fruit set, initiation of ripening, cell division, cell expansion, and fruit softening (Saure 2005; Yu et al. 2006). The content of soluble sugars increased in fruits from plants treated with eggshell powder treatments, and the highest increase, up to 24%, was obtained with eggshell powder at 60 g, as compared to the control. However, Haleema et al. (2020) reported an increase in calcium concentration followed by a reduction in soluble sugar content in tomatoes. Fruits from plants treated with calcium nitrate at 10 g exhibited the highest content of phenolic compounds.

In contrast, those from plants treated with 60 g of bone powder had the highest flavonoid levels. Increases in phenolic content under calcium nitrate treatments have been reported in other related crops, such as pepper and apple (Flores et al. 2015; Akladious and Mohamed 2018; Azam et al. 2021). The increase in phenolic compounds improves the nutritional value of aubergine fruits. In addition, calcium intake improved the formation of fruit cell wall structure and minimized the activity of polyphenol oxidase, thereby increasing the content of phenolic compounds (Kou et al. 2015). Moreover, the presence of calcium in the soil enhances the availability of phosphorus and potassium, which are essential nutrients for fruit production (Bosompem et al. 2023). The high accumulation of flavonoids in fruits from plants treated with 60 g of bone powder may be in response to the increase in osmotic pressure induced by this treatment (Hocking et al. 2016).

Independent of the calcium application source, an increase in fruit weight loss was observed during storage. This increase may be attributed to differences in fruit physiological maturity at harvest collected among the plots. According to Zaro et al. (2014), the fruit maturity stage at harvest significantly influences postharvest shelf life during storage. Also, differences in average fruit weight‐loss percentages across treatments could be due to differences in morpho‐anatomical characteristics (Bondada and Keller 2012). On the other hand, fruit firmness showed an irregular evolution during storage with both treatments; meanwhile, this physical characteristic was better maintained in fruits from plants treated with calcium nitrate at 15 g than in those with no fertilization. The low losses in fruit firmness and weight from plants treated with eggshell powder allowed for longer acceptable visual quality and, at the same time, slowed their shrivelling. Our results agree with those reported by Huang et al. (2021) for kiwi fruit. The authors showed that in firmer fruit, more water loss may be required to distort rigid tissues, whereas in softer fruit, some degree of water loss may be absorbed within the fruit before shrivelling occurs.

The degree of fruit decay is an essential criterion for both sellers and consumers in determining fruit acceptance (Bayogan et al. 2017). Fruits from plants treated with eggshell powder at 60 g treatment showed an increase in their shelf‐life up to 30 days. Fruits from plants treated with bone powder at 30 g were more susceptible to degradation during storage compared to the other treatments. Mineral analysis of the bone powder revealed relatively low calcium (Ca) concentrations but comparatively high sodium (Na) levels. Elevated sodium concentrations can accelerate postharvest fruit deterioration by inducing osmotic stress and ion toxicity, which disrupt cellular membrane integrity, impair metabolic balance, and promote faster physiological breakdown of fruit tissues (Zhu 2001; Munns and Tester 2008). Excess Na accumulation may also enhance membrane permeability and increase oxidative stress, thereby contributing to greater moisture loss and reduced storage quality (Parida and Das 2005). In contrast, calcium supplied through eggshell powder and calcium nitrate is well known to stabilize cell walls and plasma membranes by strengthening pectin cross‐linking within the middle lamella, ultimately reducing respiration rate, delaying senescence, and minimizing postharvest weight loss (White and Broadley 2003). Consequently, Ca‐rich amendments generally improve fruit firmness and storage longevity more effectively than Na‐dominant mineral sources.

## 5 Conclusion

This study aimed to assess the comparative effects of applying organic and mineral calcium sources on aubergine production, quality, and storage. Compared with mineral calcium fertilization and calcium nitrate, eggshell powder treatment improved yield per hectare. It maintained fruit length, whereas the bone powder treatment significantly increased fruit number and reduced fruit girth and size. Both fertilizers reduced aubergine fruit weight compared to the control with no fertilization. Overall, the fertilizers significantly improved the biomineral composition of fruits at harvest. In addition, among the fruits from the different treatments, those from plants treated with eggshell powder (60 g) exhibited better visual quality characteristics and longer shelf life. Hence, the use of eggshell and beef bone powders as calcium sources can improve aubergine fruit production, quality, and storage, while providing an alternative to mineral calcium.

## Author Contributions

**Batoum Yvan Cedric:** methodology, software, formal analysis. **Djabou Mouafi Astride Stéphanie:** conceptualization, writing – original draft, writing – review and editing, methodology, data curation. **Kouam Eric Bertrand:** writing – review and editing, supervision. **Niemenak Nicolas:** writing – review and editing, supervision, resources. **Boutchouang Pouengue Rodrigue:** writing – review and editing, software, formal analysis, methodology.

## Funding

The authors have nothing to report.

## Conflicts of Interest

The authors declare no conflicts of interest.

## Data Availability

The data that support the findings of this study are available from the corresponding author upon reasonable request.
